# Genome architecture reveals hidden strain-level diversity in the highly conserved fish pathogen Nocardia seriolae

**DOI:** 10.1099/acmi.0.000908.v4

**Published:** 2026-05-19

**Authors:** Sk Injamamul Islam, Khandker Shahed, Md Imtiaz Ahamed, Haitham Mohammed

**Affiliations:** 1 BioMac Lab, Dhaka, Bangladesh; 2 Department of Rangeland, Wildlife and Fisheries Management, Texas A&M University, College Station, Texas, USA

**Keywords:** bioinformatics, fish, *Nocardia*, pangenome, pathogen, virulence

## Abstract

Fish nocardiosis, caused by *Nocardia seriolae*, poses a persistent threat to the aquaculture industry. Yet, the genomic determinants underlying strain-level diversity and adaptation remain poorly understood due to the high nucleotide conservation of this species. The objective of this study was to characterize genome-level variation among all publicly available complete genomes using integrative comparative genomics approaches that extend beyond nucleotide identity metrics. Nine complete genomes were analysed using average nucleotide identity, whole-genome structural rearrangement analysis, single-copy phylogenomics, genomic island prediction, pangenome reconstruction, functional annotation and antimicrobial resistance and virulence profiling. Although average nucleotide identity values confirmed extreme nucleotide conservation across all strains, extensive strain-specific structural rearrangements, including inversions, translocations and duplications, were detected. Phylogenomic reconstruction resolved geographically associated lineages despite minimal nucleotide divergence. Pangenome analyses supported an open pangenome dominated by a large, conserved core genome with limited but persistent accessory gene content and core biased gene duplication. Functional profiling revealed enrichment of transcriptional and metabolic processes, while resistance and virulence analyses identified only conserved intrinsic determinants shared across all strains. These findings demonstrate that genome architecture and pangenome dynamics provide critical resolution for understanding *N. seriolae* diversification. The study highlights the importance of integrating structural genomics and phylogenomics for strain tracking and surveillance in aquaculture systems.

## Data Summary

All the data presented in the study were accessed in the NCBI database with accession no. GCA_033097305.1, GCA_030562925.1, GCA_033097325.1, GCA_031852375.1, GCA_002356035.1, GCA_001865855.1 and GCA_018223685.1. Supplementary materials can be found in Figshare, DOI: https://doi.org/10.6084/m9.figshare.26948269.v1 [1]. All bioinformatics analyses in this study were conducted using publicly available tools as described in the Methods section. The custom code used for data analysis is publicly available on FigShare (https://doi.org/10.6084/m9.figshare.31877236).

## Introduction

*Nocardia seriolae*, a Gram-positive pathogen that resides within cells, infects fish with compromised immune systems or damaged surfaces, resulting in significant losses in aquaculture operations [2]. The identified pathogens causing fish nocardiosis are *Nocardia salmonicida*, *Nocardia asteroides* and *N. seriolae* [3]. Over the last three decades, several studies have consistently shown that *N. seriolae* has emerged as the primary etiological agent responsible for fish nocardiosis [4,6]. *N. seriolae* infection seldom leads to sudden and widespread fish mortality; the typical incidence rate is between 15 and 30%, but in difficult situations, the cumulative mortality can reach 100% [7]. Currently, the number of cases of *N. seriolae* infection in East and Southeast Asia is steadily growing each year [8,9]. Additionally, the number of fish species susceptible to *N. seriolae* infection is also expanding, resulting in substantial consequences for the affected aquaculture industries [3]. Despite causing significant economic losses in fish farming worldwide, efficient strategies to combat nocardiosis are still lacking [8].

Despite the significant damage caused by *N. seriolae* to fish culture, its pathogenic mechanism remains poorly understood. Prior research has shown that *Nocardia* spp. possesses highly effective mechanisms to resist elimination by phagocytes [10]. During the infection phase of *N. seriolae*, the bacterium can survive inside the macrophages after being engulfed. This survival ability is likely attributed to its immune evasion mechanism and the complex structure of its cell wall [11]. *N. seriolae* that survive within phagocytes can persistently infect various fish organs by migrating via the macrophages, proliferating and continuously infecting the host. To counteract the invasion and dissemination of *N. seriolae*, the host’s immune system mobilizes immune cells and epithelial cells to encircle the site of infection, creating a granulomatous tissue reaction. However, the presence of *N. seriolae* in the centre of the granuloma, coupled with the diffusion barrier formed by mycolic acid in its cell wall, reduces the effectiveness of antibiotics and other chemical medications. Furthermore, the granuloma may serve as a reservoir for *N. seriolae*, enabling the infection’s persistence and contributing to the prolonged course of the disease [12].

Recently, advancements in complete genome sequencing technology have significantly enhanced the accuracy and effectiveness of phylogenetic analysis, genotyping, bacterial identification, evaluation of antibiotic resistance genes and disease monitoring [13,14]. Previous investigations have examined 7 complete genomes and 17 partial genomes of *N. seriolae* to verify their accuracy and determine the complete set of virulence factors they possess. However, to the authors’ knowledge, a comprehensive pangenome analysis of all complete *N. seriolae* sequences has not yet been conducted. Considering the rapid advancement of high-throughput sequencing technology, it is now possible to quickly acquire hundreds to thousands of distinct sample genomes.

Pangenomic analysis is a powerful approach for studying the distribution of gene families within populations. It can be paired with functional annotation to reveal the functional variations of genes across different groups. Tools for bacterial pangenome analysis are continuously being updated and refined [15]. Comparative pangenomics has been increasingly applied as an exploratory framework to characterize genomic diversity and functional potential across related bacterial pathogens [16]. In this study, we focused on the complete genome sequences of *N. seriolae* strains with available high-quality assemblies and documented relevance to aquaculture and research. These genomes were retrieved from the National Center for Biotechnology Information (NCBI) database and analysed using comparative pangenomic approaches to describe patterns of genome conservation, structural variation and gene content diversity within the species.

Rather than providing definitive resolution of pathogenic mechanisms or evolutionary trajectories, the objective of this work was to generate a genomic baseline that highlights conserved and variable genomic features among *N. seriolae* strains. Specifically, we aimed to identify core and accessory gene components, assess genome architecture variability and explore how structural genomic features may contribute to pangenome dynamics. The results of this study provide a foundation for future functional, experimental and epidemiological investigations into *N. seriolae* diversity, pathogenicity and adaptation, while acknowledging the inherent limitations of genome-based inference alone.

## Methods

### Genome retrieval, quality control and comparison

A total of nine complete genome sequences of *N. seriolae* isolated from fish hosts were retrieved from the NCBI database (https://www.ncbi.nlm.nih.gov/) (accessed November 2025). For each genome, associated metadata and BioSample records were also obtained. Table 1 summarizes the accession numbers and strain information included in this study. Only complete, closed genomes were included, while chromosome-level, scaffold-level and contig-based assemblies were excluded to minimize assembly-related biases in downstream analyses [17]. Complete genomes provide accurate gene boundaries, reliable orthogroup inference and robust core–accessory genome partitioning, thereby reducing errors caused by gene fragmentation and misannotation that commonly affect draft assemblies [18]. This strategy improves the reliability of pangenomic, phylogenomic and virulence gene analyses, consistent with established best practices in bacterial comparative genomics. In addition, the genomes were re-annotated using the Prokka v1.14.6 (Prokaryotic genome annotation tool) with default parameters [19]. To further refine the data, the CheckM v1.2.1 [20] was employed for genome quality filtration. The criteria for genome completeness were set at a minimum of 90%, while the maximum allowed contamination was 5%. In addition, the genomes were evaluated for their average nucleotide identity (ANI) using FastANI v1.33 [21] and ANIclustermap [22]. FastANI is specifically designed to quickly calculate the ANI between whole genomes without relying on alignment. ANI represents the ANI between orthologous gene pairs that are present in two different microbial genomes. Furthermore, Synteny and Rearrangement Identifier (SyRI) v1.6 [23] was implemented to identify and characterize structural genomic rearrangements among *N. seriolae* genomes using whole-genome assembly alignments. SyRI detects syntenic regions and infers large-scale structural variants, including inversions, translocations and duplications, as well as local sequence variations within syntenic and rearranged regions.

**Table 1.** Details of the complete genomes of *N. seriolae* strains analysed in this study

Strain	Assembly	Level	Host fish species	Size (Mbp)	G+C (mol%)	CDS	Location
KGN1266	GCA_033097305.1	Complete	Japanese amberjack	8.22251	68.1	8,045	Japan
20230510	GCA_030562925.1	Complete	Mandarin	8.12311	68.1	7,335	China
24013	GCA_033097325.1	Complete	Japanese amberjack	8.11321	68.1	7,815	Japan
SDAT 0011	GCA_031852375.1	Complete	Snakehead	8.28384	68.1	7,545	China
UTF1	GCA_002356035.1	Complete	Japanese amberjack	8.12173	68.1	7,293	Japan
EM150506	GCA_001865855.1	Complete	Japanese eel	8.30452	68.1	7,544	South Korea
TL20	GCA_018223685.1	Complete	Largemouth bass	8.29881	68.1	7,466	China
AHLQ20-01	GCA_052059615.1	Complete	Largemouth bass	8.129	68	7,268	China
NS01	GCA_037478025.1	Complete	Largemouth bass	8.1	68	7,366	China

### Phylogenomic study of the genomes

Orthofinder v2.5.5 [24] and OrthoVenn3 (https://orthovenn3.bioinfotoolkits.net/) [25] were utilized to identify single-copy genes across the genomes for the phylogenomic analysis. This process involved comparing all genomes against each other using DIAMOND v2.0.14 [26]. This bioinformatic tool leverages the Markov Clustering algorithm, implemented in the MCL program, to make these predictions [27]. The phylogenomic tree of the species was then constructed with the assistance of MAFFT and FastTree [28,30], based on the identified single-copy genes shared among different strains. FastTree estimates trees under an approximate maximum likelihood framework based on a generalized time reversible substitution model, with branch support assessed using the Shimodaira–Hasegawa procedure. This phylogenomic tree reflects evolutionary relationships grounded in the concept of evolutionary time. The construction of all phylogenomic trees was carried out using the web services of Interactive Tree Of Life (iTOL) v5.5.8 (https://itol.embl.de/) [31].

### Genomic islands prediction and synteny analysis

To predict genomic islands (GIs) within the complete genome sequences, the IslandViewer4 (http://www.pathogenomics.sfu.ca/islandviewer/) was used [31]. IslandViewer4, an integrated platform that combines multiple sequence composition and comparative genomics approaches to identify putative horizontally acquired regions. This tool enables reliable detection and visualization of GIs, which often contain genes associated with adaptive functions such as virulence, host interaction and environmental persistence [31]. In parallel, the blast Ring Image Generator software v0.95 [32] was used to perform whole-genome comparative analyses of all nine *N. seriolae* genomes, allowing visualization of genome-wide similarity patterns and identification of conserved and variable regions across strains. In addition, gene synteny analysis was performed using the Mauve program [33] and its progressive Mauve algorithm to detect potential gene rearrangement events. Mauve identifies conserved collinear regions across genomes, allowing visualization and assessment of structural variation and syntenic relationships among the analysed *N. seriolae* strains.

### Pangenome development

The pangenome was analysed using Heap’s Law in conjunction with the micropan v2.1 package in R programming [32] and the PGAP2 (Pan-Genome Analysis Pipeline 2) [33]. Pangenome dynamics were evaluated using PGAP2 by quantifying changes in core and accessory gene content based on gene presence and absence matrices across genomes. Heaps' law was applied within this framework to estimate pangenome openness and to determine the rate of novel gene acquisition with the sequential addition of genomes. The complete pangenome for the entire dataset of genomes was generated using Anvi’o v8 [34] by following the pangenomics procedure described in this guide: https://merenlab.org/2016/11/08/pangenomics-v2/ . In brief, the following scripts were executed: ‘anvi-gen-contigs-database’, utilized to create a database, with Prodigal v2.6.3 [35] for identifying open reading frames in contigs, and ‘anvi-run-ncbi-cogs’, which provided gene annotations by utilizing the NCBI Clusters of Orthologous Groups (COG) database [36]. The genome database was created using ‘anvi-gen-genomes-storage’ and ‘anvi-pan-genome’ for visualization. Anvi’o employs the DIAMOND tool to compute the similarity between each amino acid sequence in every genome and all other amino acid sequences across all genomes in the dataset and then uses the MCL algorithm [37] to detect clusters in the results of amino acid sequence similarity.

### Functional annotation and gene encoding factor identification

The entire genome, including repetitive elements, was annotated using the eggNOG-mapper v2 [38] and COG of Proteins [39]. Sequence alignment involved mapping each sequence using either the Hidden Markov Model or DIAMOND against the eggNOG database. The optimal matching sequence of each target sequence was then classified based on its taxonomy and further categorized and annotated using COG functional categories [40]. Gene screening for virulence and antimicrobial resistance determinants was performed using ABRicate version 1.0.1 [41,42], which identifies known functional genes by mapping genome sequences to curated reference databases. Searches were conducted against the NCBI AMRFinderPlus [43], the virulence factor database (VFDB) [44], ResFinder v4.0 [45] and the comprehensive antibiotic resistance database (CARD) [46] to detect annotated virulence and antimicrobial resistance genes across all *N. seriolae* genomes. Detection thresholds were set at 80% minimum identity and 30% minimum coverage to ensure confident gene annotation and comparative assessment across strains.

## Results

### Nuclear identity and genomic re-arrangement

**Fig. 1.**
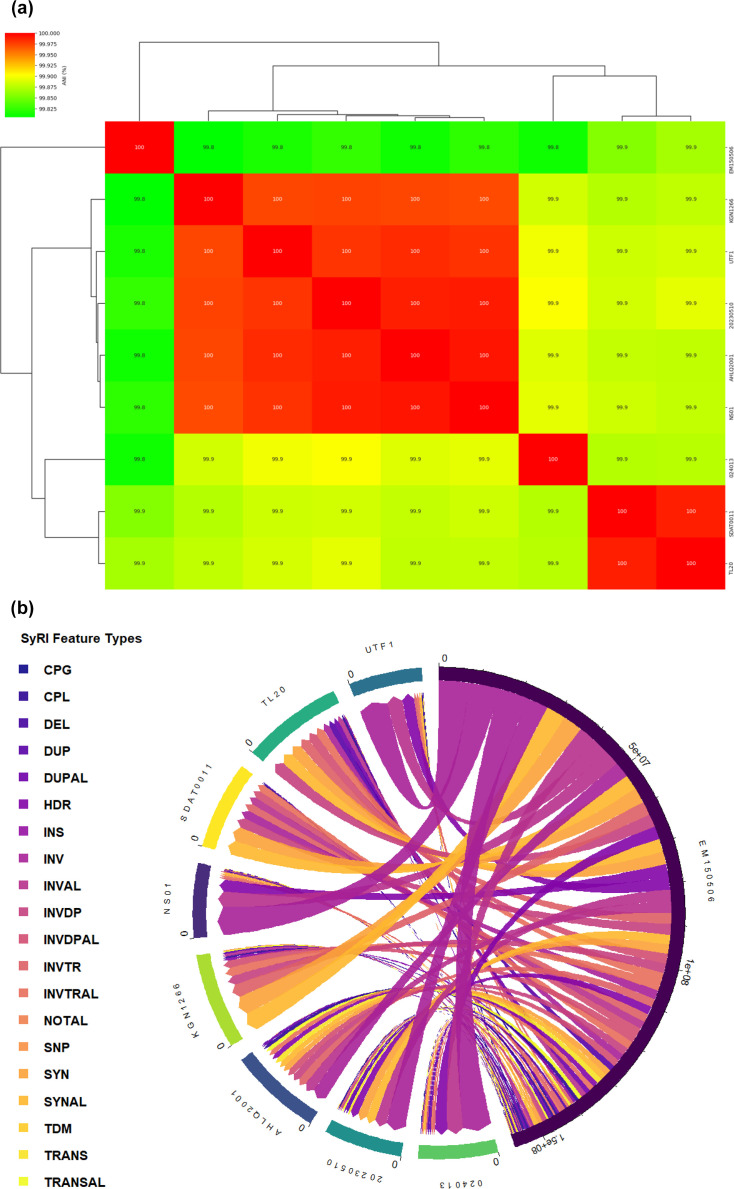
(a) ANI analysis of *N. seriolae* genomes. Heatmap and hierarchical clustering of pairwise ANI values among the nine complete *N. seriolae* genomes. Colour intensity represents the percentage of nucleotide identity between genome pairs, and clustering reflects overall genomic similarity. (b) Whole-genome structural rearrangements identified using EM150506 as the reference genome, illustrating copy number gain (CPG), copy number loss (CPL), deletion (DEL), duplication (DUP), duplicated and aligned regions (DUPAL), highly diverged regions (HDR), insertion (INS), inversion (INV), inverted and aligned regions (INVAL), inverted duplication (INVDP), inverted duplicated and aligned regions (INVDPAL), inverted translocation (INVTR), inverted translocated and aligned regions (INVTRAL), not aligned regions (NOTAL), single nucleotide polymorphisms (SNP), syntenic regions (SYN), syntenic and aligned regions (SYNAL), tandem duplication (TDM), translocation (TRANS) and translocated and aligned regions (TRANSAL) across all strains.

ANI analysis revealed a very high genomic similarity among the nine *N. seriolae* strains, with pairwise ANI values ranging from ~99.8 to 100% (Fig. 1a). Hierarchical clustering based on ANI separated the strains into two major groups, with one group comprising EM150506, KGN1266, UTF1, 20230510, AHLQ2001 and NS01, and a second group comprising 024013, SDAT0011 and TL20. Although this clustering suggests subtle population structure, overall divergence remained minimal, confirming species-level relatedness while indicating that ANI alone provides limited resolution for detecting strain-specific genomic differences within this dataset.

To achieve higher-resolution insight into genome-level variation, whole-genome structural rearrangements were systematically quantified using SyRI, with EM150506 designated as the reference genome. The strain EM150506 was selected as the reference genome due to its status as one of the earliest complete and well-annotated genomes of *N. seriolae*, as well as its relatively divergent phylogenetic position, which enhances the detection of structural genomic variation across strains. A comprehensive summary of all SyRI-detected features, including syntenic regions, inversions, translocations, duplications, insertions, deletions and unaligned segments, was compiled into File S1, available in the online Supplementary Material. SyRI analysis revealed extensive conservation of genome synteny across all strains relative to the reference genome EM150506, consistent with the high ANI values. However, strain-specific structural variation was clearly evident when SyRI features were compared using event counts alone (Fig. 1b; File S1). The number of syntenic regions varied markedly among strains, ranging from 4 to 5 regions in strains 024013, NS01 and UTF1 to 107 regions in strain KGN1266, indicating substantial differences in genome collinearity relative to the reference. Inversion events were unevenly distributed across strains. Strains 024013 and NS01 each contained a single inversion event, whereas strains AHLQ2001, 20230510, KGN1266, SDAT0011 and TL20 exhibited between five and eight inversion events, indicating lineage-specific genome reorientation. Translocation counts showed pronounced variation, with 5 events detected in strains 024013 and NS01, increasing to 10 in 20230510, 11 in AHLQ2001, 16 in TL20, 17 in SDAT0011, 19 in UTF1 and reaching a maximum of 29 translocations in KGN1266. Duplications also contributed to strain-level differences, with duplication counts ranging from 8 events in strain 024013 to more than 20 events in several strains, including NS01, UTF1 and KGN1266. Insertion and deletion events further distinguished genome architectures among strains, with SDAT0011 and TL20 exhibiting higher numbers of these events relative to other genomes. Highly diverged regions were detected in all strains, but their counts varied substantially, indicating localized sequence divergence independent of overall nucleotide similarity. Tandem repeat events were present at low and relatively consistent frequencies across strains, contributing minimally to overall structural variation. Overall, these results demonstrate that despite extremely high nucleotide-level similarity observed in the ANI analysis, *N. seriolae* strains differ substantially in the number and type of structural genomic rearrangements. The strain-specific distribution of SyRI-detected events shows that genomic diversity within this species is primarily reflected in genome architecture rather than nucleotide substitutions alone.

### Single-copy phylogenomic study

Single-copy orthogroup analysis provided a high-resolution framework to resolve the evolutionary relationships among the nine strains. OrthoFinder classified protein-coding genes from all complete genomes into orthogroups and identified a large set of conserved single-copy genes shared across strains. These single-copy orthogroups were used to reconstruct the phylogenomic relationships, minimizing the confounding effects of paralogs and genome rearrangements.

**Fig. 2.**
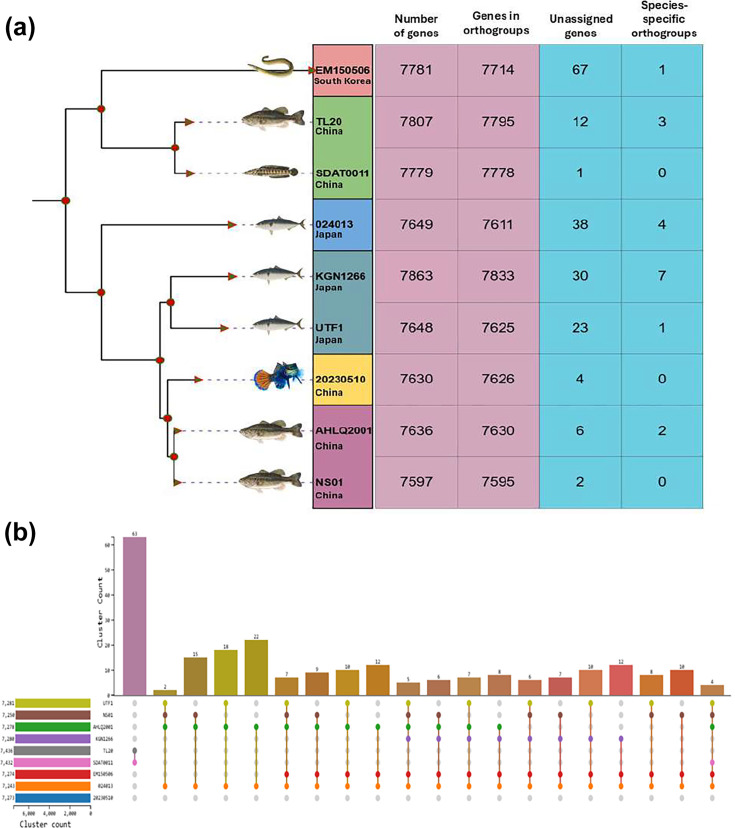
(a) Phylogenomic tree inferred from concatenated single-copy orthogroups identified across nine complete *N. seriolae* genomes using OrthoFinder. The tree illustrates the evolutionary relationships among strains, with branch lengths reflecting relative divergence based on conserved protein-coding genes. Adjacent to phylogeny, a summary table reports genome-level gene content statistics for each strain, including the total number of predicted genes, the number of genes assigned to orthogroups, the number of unassigned genes and the number of species-specific orthogroups. Strain-specific metadata, including isolation source and country of origin, are indicated to facilitate interpretation of geographic and host-associated clustering patterns. (b) Comparative analysis of protein orthogroup distribution across nine *N. seriolae* strains based on OrthoFinder clustering. The Upset plot summarizes the number of shared and strain-specific orthogroups, illustrating the size of the conserved core genome as well as accessory gene sets present in subsets of strains. Horizontal bars represent the total number of orthogroups contributed by each genome, while vertical bars indicate the frequency of orthogroups shared among specific combinations of strains.

The resulting phylogenomic reconstruction (Fig. 2a) revealed clear clustering patterns that were broadly consistent with geographic origin and genome content. Strains isolated from China formed a closely related cluster, including KGN1266 and UTF1 strains from Japan, indicating a shared evolutionary background. Within this group, short branch lengths and closely spaced nodes reflected limited divergence, consistent with the high genomic similarity observed in earlier analyses. In contrast, strains TL20 and SDAT0011 formed a distinct sublineage, while strain 024013 occupied a separate branch, indicating increased evolutionary divergence relative to other strains. The strain EM150506 from South Korea branched independently and appeared basal relative to several lineages, suggesting an earlier divergence within the dataset.

Genome content statistics supported these phylogenomic patterns. The total number of predicted genes ranged from 7,597 in NS01 to 7,863 in KGN1266. Most genes were assigned to orthogroups, with counts ranging from 7,595 to 7,833 genes per genome, indicating a highly conserved gene repertoire across strains. The number of unassigned genes was generally low, ranging from 1 in SDAT0011 to 67 in EM150506, suggesting limited strain-specific gene content outside shared orthogroups. Species-specific orthogroups were rare across all genomes, ranging from 0 to 7, further supporting the notion of a conserved core genome with modest lineage-specific expansion. Analysis of protein cluster distribution (Fig. 2b) across strains revealed that the majority of orthogroups were shared among all genomes, reflecting a large, conserved core gene set. A smaller number of clusters were shared among subsets of strains, indicating lineage-specific retention or loss of genes. These partially shared clusters were more frequent in strains showing greater phylogenetic separation, such as 024013 and KGN1266, while strains within the major Chinese lineage shared a higher proportion of clusters. Only a limited number of clusters were unique to individual strains, consistent with the low number of species-specific orthogroups observed at the genome level.

### GI prediction and comparative analysis

**Fig. 3.**
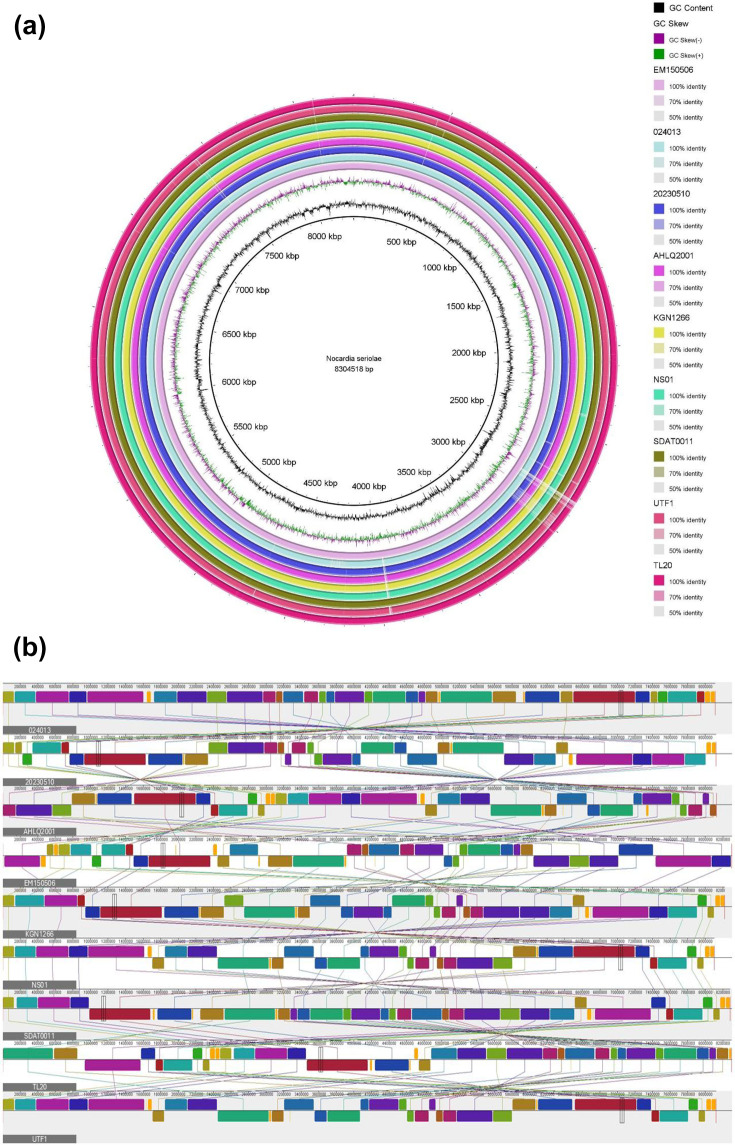
(a) Circular genome comparison illustrating whole-genome sequence similarity among nine *N. seriolae* strains using EM150506 as the reference genome. The inner tracks represent G+C content and GC skew of the reference genome, while successive outer rings correspond to individual query genomes. Shading intensity reflects different levels of sequence identity relative to the reference genome. (b) Genome-wide synteny alignment showing conserved gene order and structural rearrangements among nine *N. seriolae* genomes. Coloured blocks represent homologous genomic regions aligned across strains, while connecting lines indicate syntenic relationships between genomes. Conserved blocks reflect preservation of core genome organization, whereas inversions, translocations, insertions and deletions reveal localized genome rearrangements relative to other strains.

GI prediction was performed to investigate genome plasticity across all *N. seriolae* strains. Predicted GIs were identified using IslandViewer4 and subsequently compared across genomes to determine shared and strain-specific island content. A summary of shared and unique GIs across all strains is provided in File S1. Comparative analysis revealed that most of the predicted GIs were shared (total shared GIs 766) among multiple strains, indicating conservation of horizontally acquired regions within the species. A smaller number of GIs were strain-specific (total unique GIs 82), reflecting localized genome plasticity. These strain-specific islands were unevenly distributed across genomes, with strains exhibiting higher structural rearrangement counts also tending to harbour a greater number of unique GIs (Fig. S1). Whole-genome comparison based on sequence similarity demonstrated strong conservation across strains, consistent with the high ANI values observed previously (Fig. 3a). Circular comparative genome analysis revealed that most genomic regions exhibited high sequence identity across strains, while discrete regions showed reduced similarity or absence, corresponding to predicted GIs and non-syntenic segments. Genome synteny analysis (Fig. 3b) further supported these findings by revealing extensive conservation of gene order across genomes, interspersed with localized rearrangements. Several genomic segments displayed inversions, translocations and insertions relative to the reference genome, highlighting strain-specific genome reorganization despite overall conservation.

### Pangenome dynamics of *N. Seriolae*

**Fig. 4.**
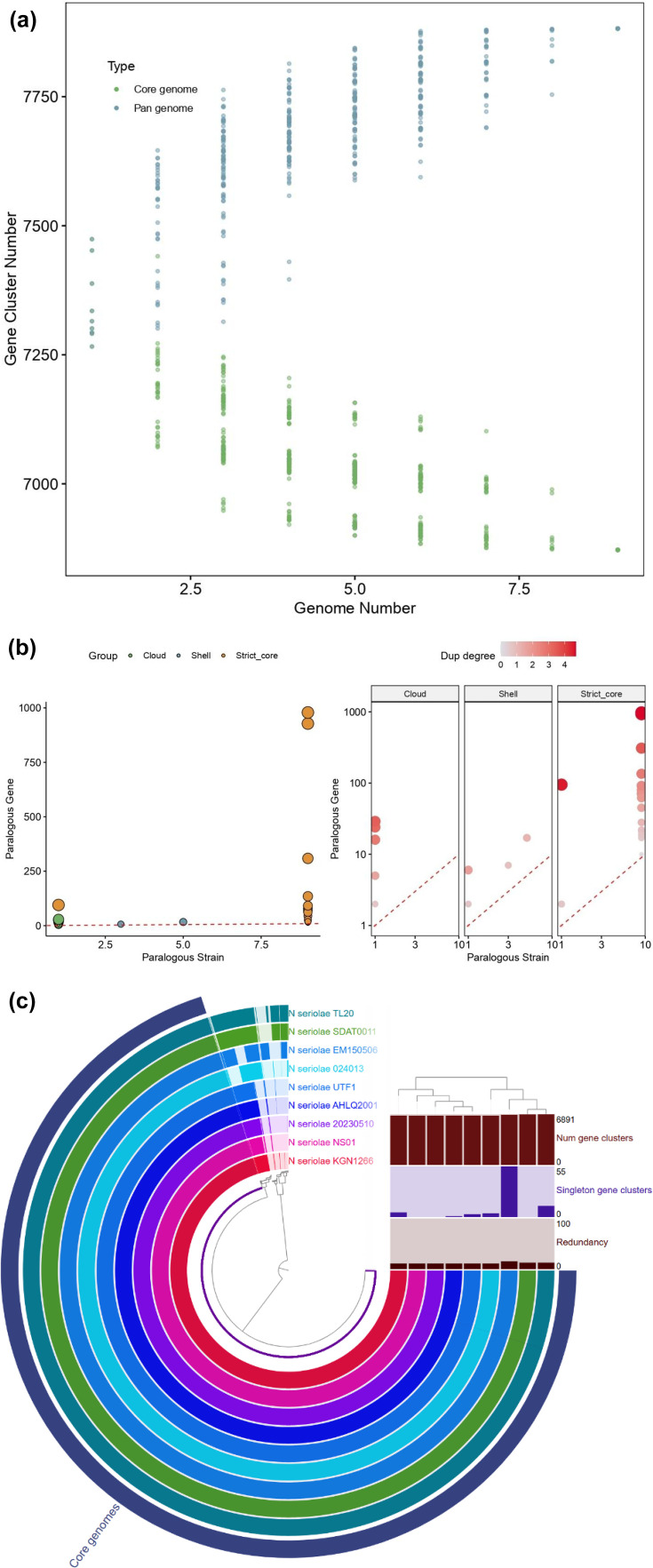
(a) Rarefaction analysis illustrates changes in pangenome size and core genome size with sequential genome addition, demonstrating continued pangenome expansion and supporting an open pangenome model. (b) Distribution of paralogous gene clusters across pangenome components, showing the relationship between paralog copy number and strain representation, with colour intensity indicating duplication degree. (c) Circular pangenome representation generated using Anvi’o showing gene cluster distribution across nine *N. seriolae* genomes. Concentric rings correspond to individual genomes, while the inner dendrogram reflects hierarchical clustering based on gene cluster presence and absence. Bar plots summarize the total number of gene clusters, singleton gene clusters and gene redundancy for each genome.

Pangenome analysis revealed a strongly conserved yet dynamically expanding gene repertoire across the analysed genomes. Gene cluster frequency analysis demonstrated that the pangenome is dominated by strict core gene clusters, defined as clusters present in all strains with a strain frequency of 1.0 (Fig. S2). These strict core genes constituted the largest fraction of the pangenome, forming a pronounced peak at a strain frequency of 1.0 and accounting for most of the total gene clusters. In addition to the strict core, a smaller number of gene clusters were classified as soft-core genes with strain frequencies between 0.95 and 0.99, representing genes that are nearly ubiquitous but absent in one or a small number of genomes. Accessory gene clusters were distributed across the shell and cloud categories. Shell genes, defined by strain frequencies between 0.15 and 0.95, formed a distinct but comparatively smaller fraction of the pangenome, indicating genes that are variably present across subsets of strains. Cloud genes, with strain frequencies between 0.0 and 0.15, represented the smallest component of the pangenome and consisted of low-frequency or strain-restricted gene clusters. The continuous distribution of gene clusters across strain frequencies, rather than discrete separation between core and accessory components, indicates ongoing gene turnover and lineage-specific gene gain and loss. Rarefaction analysis further supported this interpretation by demonstrating a continuous increase in the total number of pangenome gene clusters with the sequential addition of genomes, while the number of core gene clusters gradually decreased and approached saturation but did not fully plateau (Fig. 4a). This pattern suggests that new gene clusters are continually being introduced as additional genomes are incorporated, providing clear evidence for an open pangenome structure. Following the rarefaction analysis, the distribution of paralogous genes was examined to assess gene duplication dynamics across the pangenome (Fig. 4b). Paralogous genes were predominantly associated with the strict core genome, with several core gene clusters showing high duplication levels across strains, indicating extensive duplication of conserved genes. In contrast, shell and cloud gene clusters exhibited markedly lower paralog counts and were typically restricted to fewer strains, reflecting limited duplication among accessory genes.

Anvi’o-based pangenome analysis provided complementary insights into gene cluster distribution, redundancy and genome-specific contributions (Fig. 4c). Clustering of gene content across genomes revealed a large and stable core genome shared among all strains, consistent with the PGAP2 results. The number of gene clusters per genome was highly similar across strains, reflecting comparable genome sizes and conserved gene content. Analysis of singleton gene clusters showed that most genomes contained a low number of unique clusters, indicating limited strain-specific novelty. However, variation in singleton abundance across strains suggests differential acquisition or loss of accessory genes in specific lineages. Redundancy analysis revealed the presence of paralogous gene clusters, particularly within the core genome, indicating gene duplication events that may contribute to functional robustness or diversification. These duplicated genes were unevenly distributed among strains, suggesting lineage-specific expansion of certain functional categories. The hierarchical clustering of genomes based on gene cluster composition revealed tight grouping among most strains, reflecting the strong conservation of the pangenome structure. Subtle separation of specific lineages was also evident, consistent with patterns observed in phylogenomic and structural variation analyses.

### Functional characterization

**Fig. 5.**
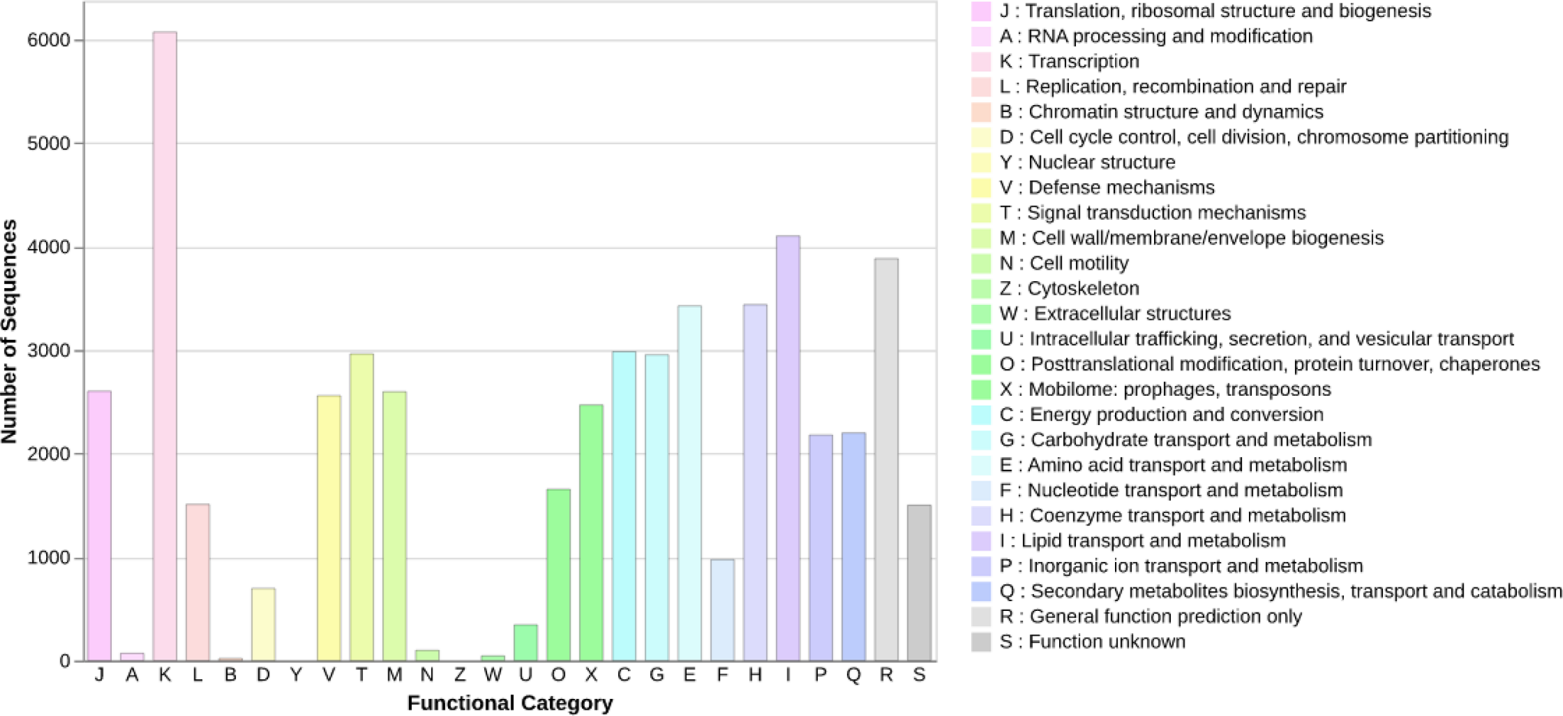
Bar plot showing the distribution of predicted protein-coding genes across COGs functional categories.

Functional annotation based on COG classification revealed a broad and conserved functional repertoire across the analysed *N. seriolae* genomes (Fig. 5). Genes were assigned to nearly all major COG functional categories, indicating functional completeness and metabolic versatility. Among the annotated categories, transcription-related genes classified under COG K represented the most abundant functional group, highlighting extensive regulatory capacity and transcriptional control within the genomes. Genes involved in translation, ribosomal structure and biogenesis under COG J were also highly represented, reflecting the strong conservation of core protein synthesis machinery. Substantial numbers of genes were assigned to replication, recombination and repair functions under COG L, as well as cell wall, membrane and envelope biogenesis under COG M, indicating active genome maintenance and structural integrity. Metabolic functions were prominently represented, with high gene counts observed for carbohydrate transport and metabolism under COG G, amino acid transport and metabolism under COG E and energy production and conversion under COG C. Genes involved in signal transduction mechanisms under COG T and intracellular trafficking, secretion and vesicular transport under COG U were consistently detected, reflecting the capacity for environmental sensing and cellular communication. Defence-related genes classified under COG V were present at moderate levels, suggesting the existence of protective mechanisms against environmental stressors or host immune pressures. In contrast, categories such as RNA processing and modification under COG A and chromatin structure and dynamics under COG B were sparsely represented, consistent with their limited roles in bacterial genomes. A notable fraction of genes was assigned to COG R, representing general function prediction only, and COG S, corresponding to genes with unknown function.

Antimicrobial resistance and virulence gene profiling using ABRicate identified a limited and conserved set of genes across all *N. seriolae* genomes (Table 2). Two genes associated with rifamycin resistance, rpoB2 and RbpA, were detected using the CARD database with high sequence coverage, indicating the presence of intrinsic rifamycin resistance related to core transcriptional machinery. In addition, two virulence-associated genes, icl and mbtH, were identified using the VFDB and are involved in metabolic adaptation and iron acquisition, respectively. All detected genes were present across all strains and formed part of the core genome, indicating conserved resistance and virulence-related functions rather than strain-specific acquisition.

**Table 2.** Summary of antimicrobial resistance and virulence genes identified using ABRicate

Gene	Identity (%)	Coverage (%)	Database	Resistance
rpoB2	81.11	98.77	CARD	Rifamycin
RbpA	81.13	92.17	CARD	Rifamycin
icl	81.07	99.77	VFDB	na
mbtH	81.6	98.15	VFDB	na

NA, not applicable.

## Discussion

Fish nocardiosis caused by *N. seriolae* is a chronic granulomatous disease responsible for substantial economic losses in aquaculture systems worldwide, particularly in Asia [47,48]. Outbreaks have been reported in multiple cultured fish species, and the pathogen is characterized by slow disease progression, intracellular persistence and difficulty of treatment once infection is established [4,49]. Although *N. seriolae* is generally considered genetically conserved, comprehensive genome-wide analyses incorporating structural variation, pangenome dynamics and functional annotation have remained scarce. The present study addresses this gap by integrating complete genome-based comparative genomics, providing a high-resolution view of genomic conservation and divergence across nine complete strains isolated from different geographic regions and hosts.

ANI analysis revealed that all nine strains exhibited very high pairwise similarity values, ranging from ~99.8 to 100%. These results are consistent with previous studies reporting extremely high nucleotide similarity among *N. seriolae* isolates from different outbreaks and geographic regions [50,52]. Hierarchical clustering based on ANI separated the strains into two subtle groups, with EM150506, KGN1266, UTF1, 20230510, AHLQ2001 and NS01 clustering together, while 024013, SDAT0011 and TL20 formed a second group. However, the minimal divergence observed highlights the limited discriminatory power of ANI for resolving strain-level diversity in this species [53]. Integration of whole-genome structural rearrangement analysis revealed pronounced strain-specific genomic variation that could not be resolved using ANI-based comparisons alone. Although extensive syntenic conservation was observed across all genomes, the number of syntenic regions varied markedly, from as few as 4 to 5 regions in strains 024013, NS01 and UTF1 to 107 regions in strain KGN1266. Structural rearrangement counts further distinguished strains, with translocation events ranging from 5 in 024013 and NS01 to 29 in KGN1266 and inversion events ranging from a single event in 024013 and NS01 to as many as 8 events in AHLQ2001, 20230510, KGN1266, SDAT0011 and TL20. These findings demonstrate that genomic diversity in *N. seriolae* is primarily expressed through genome architecture rather than nucleotide substitutions, a pattern also reported in other actinomycetes and slow-evolving intracellular pathogens [21,54].

The single-copy phylogenomic analysis revealed a clear but shallow population structure among the nine *N. seriolae* strains, which broadly reflected their geographic origin. The Chinese isolates formed two closely related subclades: one comprising TL20 and SDAT0011, which clustered tightly together, and another including 20230510, AHLQ2001 and NS01, characterized by very short branch lengths indicative of minimal divergence. In contrast, the Japanese isolates KGN1266, UTF1 and 024013 grouped separately from the Chinese lineages, with KGN1266 and UTF1 clustering closely and 024013 occupying a more distinct branch. Despite near-identical ANI values, the separation of Chinese and Japanese isolates in the single-copy phylogeny reflects regional population structuring following local dissemination within aquaculture systems [55]. The South Korean isolate EM150506 branched basally relative to both Chinese and Japanese groups, suggesting an earlier divergence within the species. Overall, the phylogeny highlights geographically associated structuring that is not apparent from ANI values alone, despite the exceptionally high nucleotide-level similarity among all strains.

Genome content statistics supported these phylogenomic patterns. Total gene counts ranged from 7,597 in NS01 to 7,863 in KGN1266, with most genes assigned to orthogroups. Unassigned genes were rare, ranging from 1 in SDAT0011 to 67 in EM150506, and species-specific orthogroups were limited to zero to seven per genome. This strong conservation of gene content indicates that lineage differentiation is driven by structural rearrangements, duplication and regulatory variation rather than large-scale gene gain or loss, consistent with observations in other aquatic bacterial pathogens [56].

GI analysis revealed that most predicted islands were shared across strains, with a total of 766 shared GIs identified. This suggests that many horizontally acquired regions were integrated before lineage diversification [57]. In contrast, only 82 strain-specific GIs were detected, and these were unevenly distributed across genomes. Notably, strains with higher numbers of structural rearrangements, such as KGN1266, SDAT0011 and TL20, tended to harbour more unique GIs, indicating a coupling between horizontal gene acquisition and genome restructuring. This pattern mirrors findings in other bacterial pathogens, where GIs often colocalize with recombination breakpoints and contribute to adaptive potential rather than encoding classical virulence factors [58]. The predominance of shared islands in *N. seriolae* supports a relatively stable pathogenic strategy, with limited recent acquisition of novel pathogenicity determinants.

Pangenome analysis revealed a strongly conserved yet open pangenome. Strict core genes, defined as gene clusters present in all strains with a frequency of 1.0, constituted the most significant fraction of the pangenome. Shell genes with intermediate strain frequencies and cloud genes with low frequencies formed a smaller but persistent accessory gene pool. Rarefaction analysis demonstrated continued expansion of the pangenome with sequential genome addition, while the core genome approached saturation but did not fully plateau, providing clear evidence of an open pangenome structure [14].

Paralog analysis further showed that gene duplication was strongly biased towards the strict core genome, with several conserved gene clusters exhibiting high duplication levels across strains. In contrast, shell and cloud genes generally displayed low paralog counts and were restricted to fewer strains. This pattern suggests that evolutionary innovation in *N. seriolae* is primarily achieved through the duplication and modulation of conserved genes, rather than through the acquisition of large numbers of novel genes. Similar pangenome architectures have been described in *Vibrio anguillarum* [41] and *Piscirickettsia salmonis* [17], all of which occupy specialized ecological niches and exhibit strong core genome conservation.

COG-based functional annotation revealed that *N. seriolae* genomes are enriched in genes involved in transcription, translation, replication and core metabolic processes. COG K, associated with transcription, was the most abundant category, reflecting extensive regulatory capacity. High representation of COG J and COG L further underscores the conservation of protein synthesis and genome maintenance machinery. Metabolic categories, including COG C, E, G and I, were also prominent, highlighting metabolic versatility and adaptation to host-associated environments. The presence of substantial numbers of genes assigned to COG R and COG S indicates that a fraction of the genome remains poorly characterized, suggesting the existence of lineage-specific functions that are not yet functionally annotated. Comparable levels of uncharacterized genes have been reported in other actinomycetes and aquatic pathogens, emphasizing the need for functional validation studies [59,60].

Furthermore, *in silico* functional analysis identified a limited and conserved set of antimicrobial resistance and virulence-associated genes across all strains. Two genes associated with rifamycin resistance, rpoB2 and RbpA, were detected in all genomes, indicating intrinsic resistance linked to core transcriptional machinery. Such intrinsic resistance is well documented in Actinobacteria [61] and Mycobacteria [62] and does not reflect the recent acquisition of resistance determinants. Importantly, no evidence of horizontally acquired or strain-specific antimicrobial resistance genes was detected. Virulence-associated genes, including icl and mbtH, were identified in all strains and formed part of the core genome. Isocitrate lyase plays a crucial role in the glyoxylate shunt and is essential for intracellular survival and persistence, whereas MbtH is involved in siderophore-mediated iron acquisition. Their conservation across all *N. seriolae* strains suggests that pathogenicity is driven by conserved metabolic and survival mechanisms rather than specialized virulence islands, consistent with the chronic nature of fish nocardiosis [63]. Together, these findings demonstrate that *N. seriolae* exhibits extremely low nucleotide-level diversity but substantial variation in genome architecture, structural rearrangements and gene duplication patterns. This highlights the limitations of relying solely on ANI or SNP-based analyses for strain discrimination and underscores the importance of integrating structural genomics and pangenome analysis into pathogen surveillance frameworks.

From an applied perspective, the conserved nature of virulence- and resistance-associated genes suggests that diagnostic targets and therapeutic strategies may be broadly applicable across strains. However, structural variation and gene duplication may influence strain-specific fitness, host interaction and outbreak dynamics. Future studies integrating transcriptomics, proteomics and experimental infection models will be crucial for linking genomic variation to phenotypic outcomes and for developing effective strategies to manage fish nocardiosis in aquaculture systems.

## Conclusion

This study presents a comprehensive genome-based comparative framework for *N. seriolae* and reveals that nucleotide-level similarity obscures substantial strain-level diversity in genome architecture. Although all nine genomes exhibited extremely high ANI values and a largely conserved gene repertoire, genomic rearrangement analysis revealed marked differences in inversions, translocations, duplications and syntenic fragmentation relative to the reference genome, indicating that structural rearrangements are a key driver of intraspecific diversity. Single-copy phylogenomics resolved lineages consistent with geographic patterns while confirming minimal gene content divergence and the rarity of species-specific orthogroups. GI comparisons revealed predominantly shared islands, accompanied by a smaller set of unique islands, supporting localized genome plasticity. Pangenome analysis of available genomes suggests a trend towards an open pangenome, characterized by a dominant core genome and a smaller but persistent accessory gene pool. However, this observation should be interpreted with caution, given the limited number of genomes analysed. Functional annotation revealed a conserved, metabolically versatile repertoire, while bioinformatics tools identified a limited set of conserved rifamycin-associated genes and core virulence-related determinants. Overall, these findings highlight the value of integrating structural genomics with phylogenomics and pangenome analysis for resolving strain-level diversity, while emphasizing the need for expanded genome sampling to further validate pangenome dynamics in *N. seriolae*.

## Supplementary material

10.1099/acmi.0.000908.v4 Uncited Supplementary Material 1.

10.1099/acmi.0.000908.v4 Uncited Supplementary Material 2.

## Abbreviations

ANI: average nucleotide identity
CARD: comprehensive antibiotic resistance database
COG: Clusters of Orthologous Groups
GIs: genomic islands
NCBI: National Center for Biotechnology Information
SyRI: Synteny and Rearrangement Identifier
VFDB: virulence factor database
