# Supplementary material for: Genome architecture reveals hidden strain-level diversity in the highly conserved fish pathogen Nocardia seriolae
Source: Access Microbiol. 2026 May 19;8(5):000908.v4. doi: 10.1099/acmi.0.000908.v4 (PMC13186243; doi:10.1099/acmi.0.000908.v4)
Supplement: Uncited Supplementary Material 1. [file acmi-8-00908-s001.pdf]

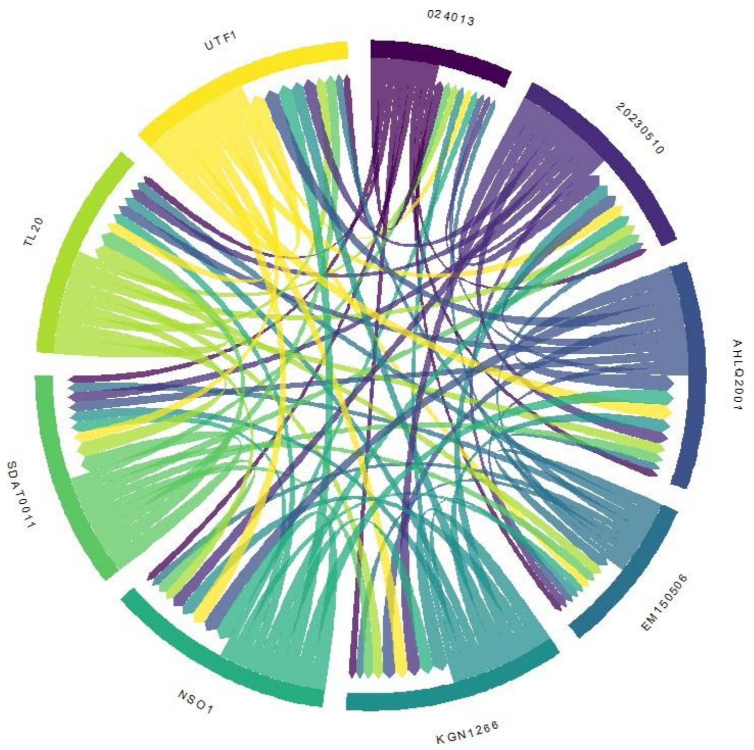

Supplementary Figure 1. Shared and strain specific genomic islands among *N. seriolae* genomes. Comparative visualization of shared and strain specific genomic islands across nine N. seriolae genomes based on genomic island prediction results. Each sector represents an individual genome, while connecting links indicate genomic islands shared between strains. The density and distribution of connections illustrate the extent of conserved horizontally acquired regions across genomes, whereas isolated links or unique segments reflect strain specific genomic islands. This figure highlights both the conserved and variable components of genome plasticity within *N. seriolae* and complements the quantitative summary provided in Supplementary File 2.

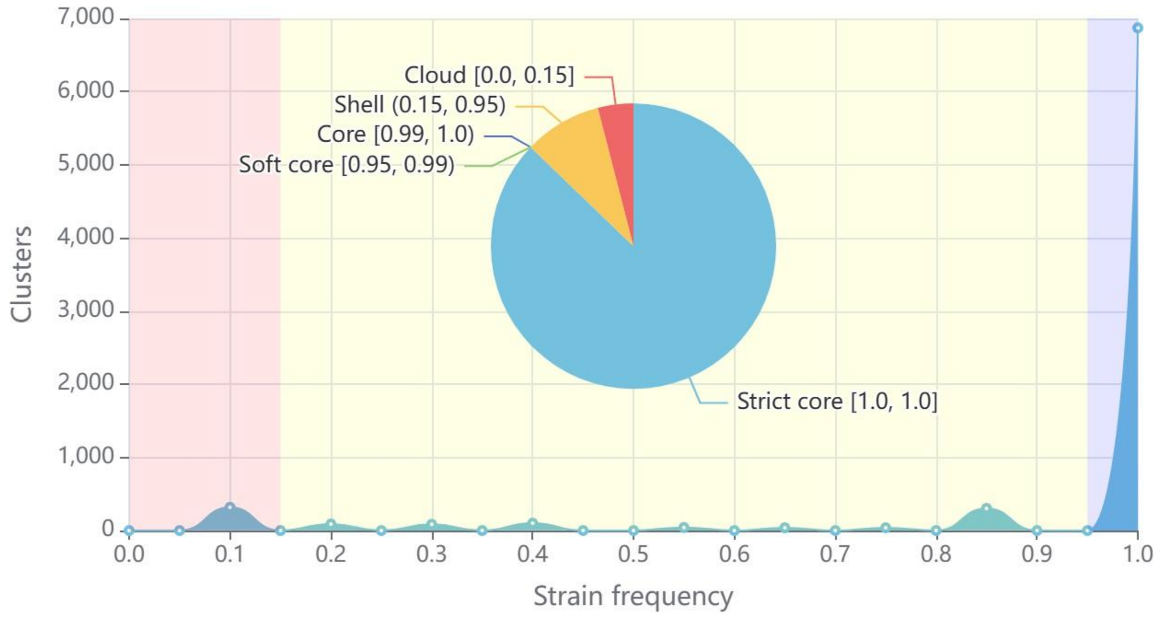

Supplementary Figure 2. Classification of gene clusters into strict core, shell, and cloud components based on their distribution across strains.
